# Oro-facial mucocutaneous manifestations of Coronavirus Disease-2019 (COVID-19): A systematic review

**DOI:** 10.1371/journal.pone.0265531

**Published:** 2022-06-01

**Authors:** Kausar Sadia Fakhruddin, Lakshman Perera Samaranayake, Borvornwut Buranawat, Hien Ngo

**Affiliations:** 1 Department of Preventive and Restorative Dentistry, University of Sharjah, Sharjah, UAE; 2 Faculty of Dentistry, The University of Hong Kong, Hong Kong Special Administrative Region, China; 3 Department of Periodontics and Implant Dentistry, Faculty of Dentistry, Thammasat University, Pathum Thani, Thailand; 4 Dental School, University of Western Australia, Nedlands, WA, Australia; International Medical University, MALAYSIA

## Abstract

We reviewed the prevalence, the likely aetiopathogenesis, and the management of oro-facial mucocutaneous manifestations of Coronavirus Disease-2019 (COVID-19), caused by the Severe Acute Respiratory Syndrome Coronavirus -2 (SARS-CoV-2). English language manuscripts searched using standard databases yielded 26 articles that met the inclusion criteria. In total, 169 cases (75 females; 94 males) from 15 countries with a spectrum of COVID-19 severities were reviewed. Gustatory perturbations were prevalent in over 70%. Mucocutaneous manifestations were reported predominantly on the tongue, palate, buccal mucosa, gingivae, and lips and included ulcers, blisters, erosions, papillary hyperplasia, macules, glossitis, and mucositis. Ulcerative lesions, present in over 50 percent, were the most common oral manifestation. Lesions resembling candidal infections, with burning mouth, were prevalent in 19%. Petechiae and angina bullosa were generally seen, subsequent to COVID-19 therapies, in 11%. Ulcerated, necrotic gingivae were documented in severely ill with poor oral hygiene. These manifestations, present across the COVID-19 disease spectrum, were commonly associated with the immunosuppressed state and/ or the concurrent antimicrobial/steroidal therapies. In summary, a wide variety of orofacial mucocutaneous lesions manifest in COVID-19. They are likely to be secondary to the disease-associated immune impairment and/or pharmaco-therapy rather than a direct result of SARS-CoV-2 infection *per se*.

## Introduction

Severe acute respiratory syndrome coronavirus 2 (SARS-CoV-2) enters the human cells *via* the angiotensin-converting enzyme II (ACE_2_) receptors and trans-membrane serine protease (TMPRSS2 and TMPRSS4) that are ubiquitously expressed in numerous tissues and organs of the body [1–3]. The oral cavity is thought to be particularly susceptible to SARS-CoV-2 infection due to the high degree of expression of ACE_2_ receptors in its mucosa lining, the tonsillar crypts, and the salivary gland epithelia [4, 5]. Therefore, it is highly likely that such readily expressed oral targets for SARS-CoV-2 infection may result in now well recognized oral manifestations such as xerostomia and dysgeusia as well as other nondescript mucocutaneous lesions that are reported in COVID-19 patients [5–7].

The array of COVID-19 manifestations in the oral mucosa is expansive and includes ulcers, blisters, enanthems, hemorrhagic lesions, and cheilitis [4, 8]. Several hypotheses have been proposed for their pathogenesis [4]. Brandão and colleagues [9] theorized that interaction between SARS-CoV-2 and ACE_2_-expressing epithelial cells of the lining mucosa could lead to a surge in the permeability and disruption of oral keratinocyte integrity and the barrier function, leading to epithelial necrosis and ulceration [9]. Others posit that thrombocytopenia, disseminated intravascular coagulation, anticoagulant therapy, or systemic inflammation could cause oral manifestations seen in COVID-19 patients [10–12]. While still others speculate the possibility of primary or secondary vascular inflammation associated with COVID-19 may be the reason for the oral lesions [12, 13]. Apart from these hypothesized reasons, there could be other factors that contribute to the loss of integrity of the oral epithelium, and these include generalized or localized immunosuppression, oral microbiome dysbiosis, and, more importantly, drug therapy [14, 15].

Despite the reported broad spectrum of orofacial manifestations of COVID-19, there are no systematic reviews, to our knowledge, that specifically address their prevalence or aetiopathogenesis. Although very recently [7] has begun a series of live systematic reviews (LSR) on oral manifestations of COVID-19, they are general in nature and do not address in detail the oral mucocutaneous manifestations. Hence, here we review the oral-mucocutaneous lesions either directly linked to SAR-CoV-2 infection or secondary to COVID-19 treatment protocols. For the sake of completion, we also review the general oral manifestations, including taste dysfunction, xerostomia, and burning mouth sensation in SARS-CoV-2, as they too are indirectly linked to mucocutaneous disease.

## Methods

### Data sources

We (LPS, KSF, and HCN) executed an electronic data search of the English language manuscripts using PubMed via OVID, SCOPUS, and Web of Science databases. Published reports between March 01, 2020, and May 01, 2021, were accessed. We identified a total of twenty-five case reports and a case series.

The Systematic review protocol was registered with the PROSPERO database (CRD42020183714).

### Study selection

**Inclusion criteria**.

- *Study design*: Case reports, case series, observational studies
- *Population*: Cases with suspected, asymptomatic, mild, moderate, or severe COVID-19 infection
- *Setting*: any healthcare setting providing consultation or treatment for COVID-19 infection (hospitals, dental clinics)
- Country or date enforced no limitations

**Exclusion criteria**.

- Review articles
- Comments, abstracts, and grey literature
- Reports presenting incomplete outcome details
- Studies evaluating gustatory dysfunction without data on oral mucocutaneous lesions
- Studies that do not meet the set study objectives

#### Search terms

A specific search string was planned for each of the databases, which included the following search terms:

SARS-CoV-2 OR COVID-19 OR Coronavirus OR novel coronavirus disease OR nCoV-19 AND oral manifestations OR oral lesions OR oro-facial manifestations OR oro-facial findings AND gustatory dysfunction OR taste disorder OR taste alteration OR dysgeusia OR hypogeusia OR ageusia AND dry mouth OR xerostomia AND glossodynia OR burning mouth syndrome (BMS) OR glossalgia AND mucosal lesion OR mucocutaneous lesion OR oral mucocutaneous lesion OR oral lesion.

#### Summary measure

The primary outcome was to review the clinical presentation of oral mucocutaneous lesions expression during SARS-CoV-2 infection, its causality, and temporal association during infection. The secondary outcome included the prevalence of gustatory dysfunctions, xerostomia, and burning mouth sensations in those presenting with oral mucocutaneous lesions.

*Electronic data search and analysis*. We followed PRISMA (Preferred Reporting Items for Systematic Reviews) guidelines for a systematic and comprehensive approach.

We examined the titles and abstracts of all relevant published reports that met our set inclusion criteria during stage one of the three-staged, electronic data-search and analysis. A full-text review of all the related articles was performed during stage two to view the data comprehensively. A thorough analysis of the full text of the retrieved literature ensured that the eligibility criteria were met and the reported outcomes were according to the set outcome measures. In addition, references of the included reports were examined as a backward search. During stage three: the reviewers (LPS and KSF) extracted and evaluated the data.

After the full-text review, specific points related to the characteristics of each included study were charted. This facilitated in classifying the setting, study design, intervention, and reporting jurisdiction. Besides, the sample size, evaluation time, assessment methods, and study conclusions were systematically examined. Finally, the third reviewer (HCN) cross-checked the data to verify its accuracy. The identified manuscripts were compiled using a bibliographic software tool, Endnote version 9 (Clarivate Analytics, USA). A summary of the characteristics of included reports is provided in (Table 1).

**Table 1:** Characteristics of the included studies with risk of bias.

Study (Study design) Country	Sample (n) Age Gender	Comorbid conditions	Oral Manifestation		Oral mucocutaneous lesions								Risk of Bias (acc. JBI)
			Gustatory dysfunction (Dysgeusia/ hypogeusia)	Xerostomia/ Others	Ulcer & erosion/ Aphthous-like lesions (ALL)	Herpetiform /zosteriform lesion	White/red plaque	Erythema multiforme-(EM)-like lesions	Petechiae & macular lesions	Vesicles and Pustules/ Angina bullosa like lesions	Necrotizing periodontal disease	Non-specific lesions (mucositis)	
**Aghazadeh N et al. 2020 (CR)** USA	N = 1 9 yrs. F	Healthy	NM	NM	**+**								**L**
**Al-Khanati et al. 2020. (CR)** Syria	N = 1 24 yrs. M	NM	NM	Burning sensation on the tongue	**+**								**M**
**Ansari R et al. 2021 (CR)** Iran	N = 2, 56 yrs. F 75 yrs. M	**Case 1:** well-controlled HT, COPD **Case 2:** HT, Diabetes, Obesity, and RF **Case 3**: Obesity, Parkinson, HT, COPD **Case 4:** Diabetes, HT **Case 5 to 8**: NCC	NM	NM	**+**								**L**
**Brandão TB et al. 2021** **(CS)** Brazil	N = 8, 28–81 yrs. F = 3; M = 5	NCC	Present in all cases except case 3 & 4	NM	**+**								**L**
**Cebeci K et al. 2020 (CR)** Turkey	N = 1 51 yrs. M	NM	Present	NM					**+**	**+**		**+**	**L**
**Chaux‑ Bodard A-G et al. 2020 (CR)** France	N = 1, 45 yrs. F	NCC	Present	NM	**+**								**M**
**Ciccarese G et al. 2021 (CR)** Italy	N = 1 19 yrs. F	Atopic person, mostly on analgesics and antibiotics prescriptions	NM	NM	**+**								**L**
**Corchuelo J et al. 2020 (CR)** Colombia	N = 1 40 yrs. F	NM	No alteration in taste	Xerostomia/Post-inflammatory pigmentation			**+**		**+**				**L**
**Cruz Tapia RO et al. 2020 (CR)** Mexico	N = 4 41–55 yrs. F = 3, M = 1	**Case 1:** NM **Case 2:** NM **Case 3:** NM **Case 4:** NM	Dysgeusia in the male patient	Burning mouth symptom in a male patient						**+**		**+**	**M**
**Díaz Rodríguez M et al. 2020 (CR)** Spain	N = 3 43–78 yrs. F = 2, M = 1	**Case 1:** NM; history of RAS **Case 2:** NM; No history of RAS **Case 3:** NM; No history of RAS	**Case 1&2**: present	**Case 1&2**: burning sensation **Case 3**: intense xerostomia		**+**							**M**
**Dominguez-Santas M et al. 2020** **(CR)** Spain	N = 4 19–43 yrs. F = 1, M = 3	HT; Renal transplant; On regular immunosuppressants and daily prophylactic cover with Enoxaparin sodium for PVT	NM	NM	**+**								**L**
**dos Santos JA et al. 2020** **(CR)** Brazil	N = 1 67 yrs. M	NCC	Hypogeusia	Extremely viscous saliva		**+**	**+**						**L**
**Favia G et al. 2021** **Observational descriptive study** Italy	N = 123 Median age 72 yrs. F = 53 M = 70	NM	Present in over 80% [Dysgeusia 64% Hypogeusia 27% Ageusia 9%]	Pain & burning symptoms. **Moderate COVID cases** (87%) **Severe COVID cases** (88%) **Critical Cases** (83%)	**+**	**+**	**+**	**+**	**+**	**+**	**+**		
**Glavina A et al. 2020** **(CR)** Croatia	N = 1 40 yrs. F	NM	Present	Pain and burning sensation		**+**	**+**					**+**	**L**
**Indu S et al. 2020** **(CR)** India	N = 1 Age (NR) M	NM	NM	NM	**+**								**M**
**Jimenez-Cauhe J et al. 2020** **(CR)** Spain	N = 3 58–77 yrs. F	Hypercholesterinemia and Coronary heart disease	NM	NM					**+**				**M**
**Kämmerer T et al. 2021** **(CR)** Germany	N = 1 46 yrs. M	NCC	NM	NM		**+**							**L**
**Kitakawa et al. 2020** **(CR)** Brazil	N = 1 20 yrs. F	NCC	NM	Severe pruritis		**+**							**L**
**Labé P et al. 2020** **(CR)** France	N = 2 6 yrs. (M) 3 yrs. (M)	Healthy	NM	NM		**+**					**+**	**+**	**M**
**Malih N et al. 2020** **(CR)** Iran	N = 1 38 yrs. M	**Case 1:** NCC **Case 2:** Diabetes and HT **Case 3:** Obesity and HT	Dysgeusia	NM	**+**								**L**
**Martín Carreras- Presas C et al. 2020** **(CR)** Spain	N = 3, 56–65 yrs. F = 1, M = 2	NCC	**Case 1:** present **Case 2 & 3:** NR	NM		**+**		**+**					**L**
**Patel J et al. 2021** **(CR)** United Kingdom	N = 1 35 yrs. F	NM	NM	NM							**+**		**L**
**Sakaida T et al. 2020** **(CR)** Japan	N = 1 52 yrs. F	Diabetes and HT	NM	NM	**+**								**L**
**Soares CD et al. 2020** **(CR)** Brazil	N = 1 42 yrs. M	History of MRS	Hypogeusia	Xerostomia	**+**							**+**	**L**
**Taşlıdere B et al. 2021** **(CR)** Turkey	N = 1 51 yrs. F	NCC	NM	NM									**L**
**Tomo S et al. 2020** **(CR)** Brazil	N = 1 37 yrs. F		Dysgeusia	Xerostomia. Burning tongue sensation								**+**	**L**

NR = Not reported; CR = Case report; CS = Case series; NCC = No chronic condition; F = female; M = male; MRS = Melkersson–Rosenthal syndrome; HT = hypertension; COPD = chronic obstructive pulmonary disease; RF = renal failure; PVT = pulmonary venous thromboembolism JBI (Joanna Briggs Institute) critical appraisal tool for CR [low risk of bias (> 70% scores); moderate risk of bias, (scores between 50% and 69%); and high risk of bias (scores were below 49%)].

### Quality and overall risk of bias assessment

During stage three, two investigators (LPS and KSF) independently performed the quality assessment of the eligible studies using the Joanna Briggs Institute Critical Appraisal Checklist for (i) case series, (ii) case reports, and (iii) analytical cross-sectional studies’ Critical appraisal checklists’ [16]. The third and fourth reviewers (HCN and BB) were referred to in case of any disagreement. The evaluated studies were documented as low-risk, moderate, or high-risk (Table 1). Case reports with a high risk of bias were excluded from the present review. All reviewers discussed decisions based on cumulative scores. The report was characterized as low risk when the ’yes’ score reached ≥70%, moderate when the score is between 50%-69%, and low when the score is ≤49%.

## Results

**Fig 1:**
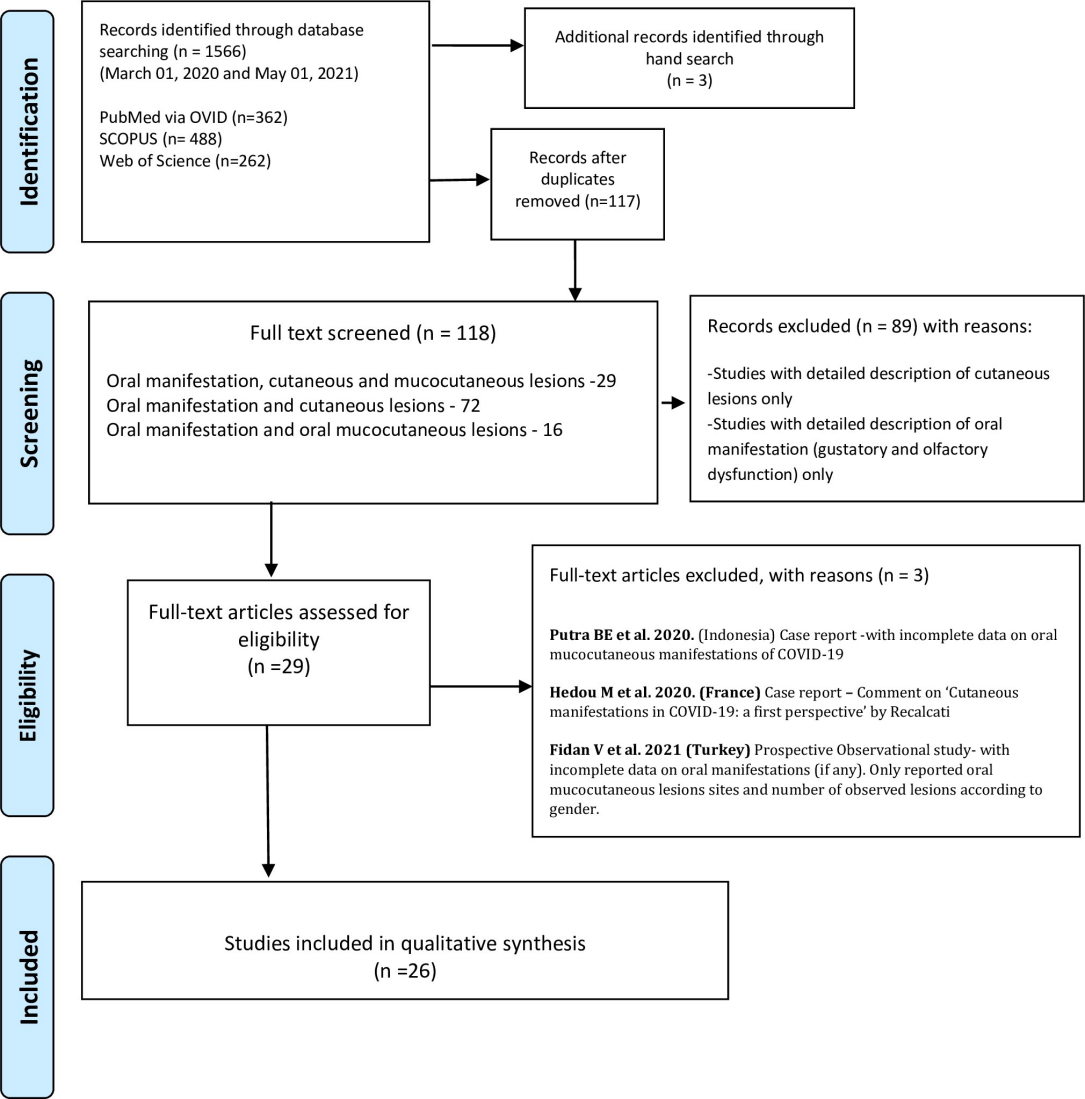
PRISMA flow chart of the literature search and study selection.

Of the 118 full texts reviewed, only 26 articles met the set inclusion criteria, Fig 1. The reviewed articles encompassed an observational study [12], a case series [9], and 24 case reports [10, 11, 13, 17–37], encompassing a total of 169 patients (75 females; 94 males) with various degrees of COVID-19 disease severities, ranging from suspected, to intensive care bound critical cases.

The largest number of reported cases were from an observational study, which included 123 COVID-19 patients with moderate, severe, and critically ill-disease severities [12]. Moreover, most of the reviewed case reports included one to four cases, while the only available case series described oral manifestations in eight COVID-19 patients [9]. Incidentally, the included case reports comprised case studies of three children aged between 3-to-9-years [17, 31] and 19-to-81-year-old adults.

Oral mucocutaneous lesions were almost equal among both females and males with 46% (n = 77) and 54% (92) representation, respectively. In several reports, single or comorbid-chronic systemic conditions such as hypertension, diabetes mellitus, obesity were prevalent (Table 1).

Related to SARS-CoV-2 infection, the spectrum of taste dysfunctions, including dysgeusia, hypogeusia, and ageusia, was prevalent in 74% (125) cases (Table 1). Symptoms of xerostomia were reported in few reviewed case reports with no gender predilection [10, 23, 24, 37]. A Brazilian case report [11] of a 67-year-old male COVID case reported extremely viscous saliva (Table 1). Additionally, we found quite a few reports with symptoms of burning mouth sensations in moderate to severe severity COVID-patients, some of which were associated with suspected candidal infection [12, 13, 18, 24, 26, 37].

**Fig 2:**
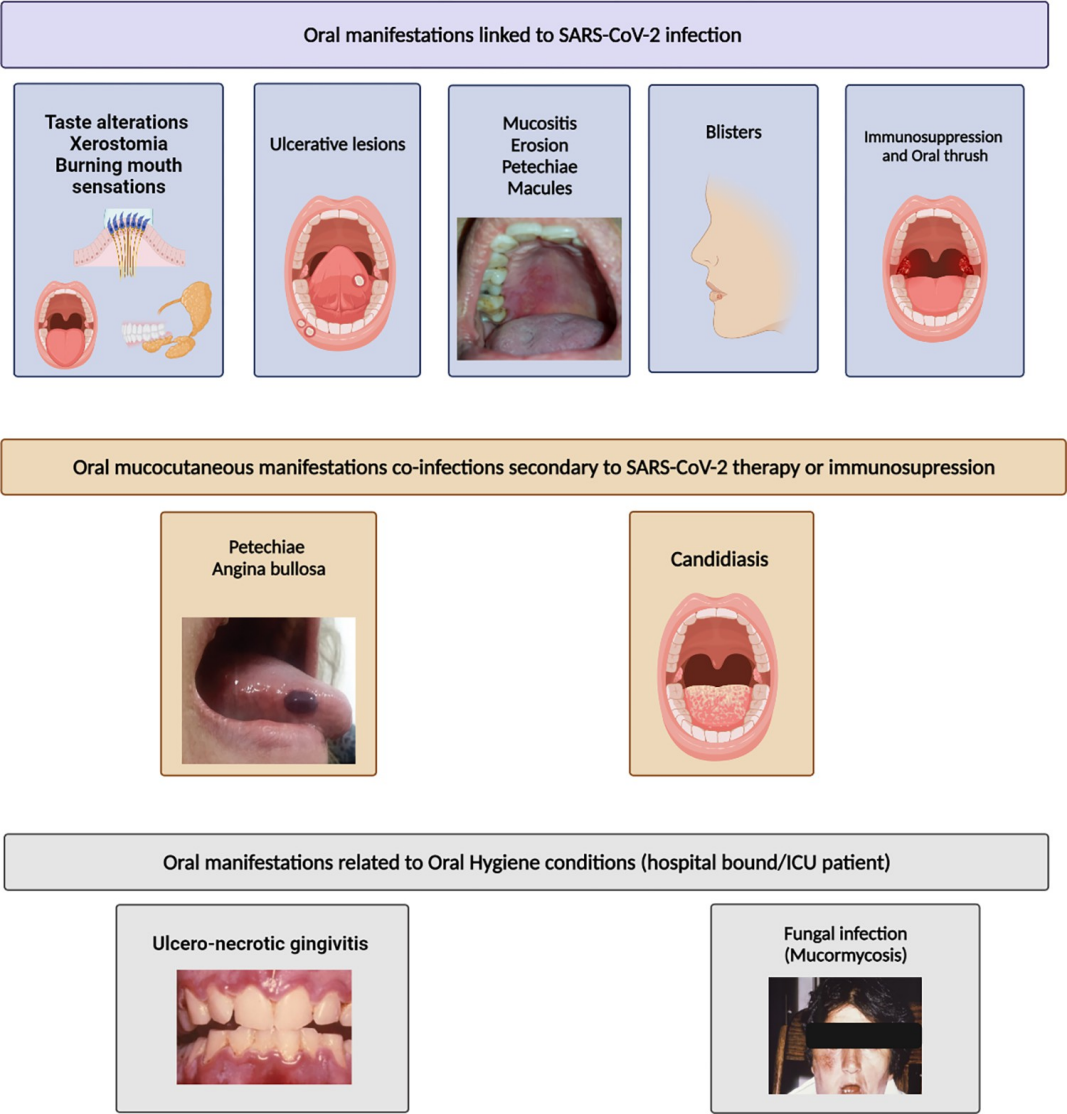


COVID-related oral mucocutaneous manifestations were varied and diverse. Painful, ulcerative lesions were the most predominant (Fig 2), present in over 50% of cases. Other manifestations included blisters, erosive lesions akin to erythema multiforme, macules, non-specific mucositis, and post-inflammatory pigmentation.

The mucosal lesions differed in quantity, size, and appearance either at single or multiple sites of the oral mucosa. There was no specific intraoral anatomical predilection for the lesion presentation, and an equal proportion of lesions were present in both the keratinized as well as the non-keratinized mucosa. For instance, a 40-years-old female with an asymptomatic COVID-19 infection presented with a painful aphthous ulcer on the attached gingiva, with multiple painless petechiae on her lower lip and a whitish area at the back of the tongue [23]. In several cases, blisters preceded ulcerative lesions [12, 26, 30, 33]. Clusters of herpetiform-like blisters progressed into erythematous ulcerative lesions followed by mild bleeding and pain, in another case [12].

Lesions either similar to or reported as candidal infections were present in 19%, some of which presented with burning oral sensation. Petechiae alone or associated with angina bullosa were expressed either prior or together with other COVID-19 symptoms in 11% [12, 20, 22, 23, 28]. Papillary hyperplasia on the dorsum and lateral borders of the tongue were also common signs observed in about 28% of the cases [12]. In addition, non-specific mucositis, including erythematous-macules and papules, pustules, and plaques, was reported in various intra-oral sites.

Other oral manifestations included fissured or de-papillated tongue, pigmentation, white/red plaques, necrotizing periodontal infection, erosive cheilitis, and spontaneous bleeding (Table 2). Interestingly, a case with a history of Melkersson–Rosenthal syndrome (MRS) had a recurrence of MRS symptoms associated with severe COVID-19 infection [36].

**Table 2:** SARS-CoV-2 related oral manifestation latency time, duration, and therapy.

Study	COVID Severity	Appearance of mucocutaneous lesions	Time to resolution of mucocutaneous lesions after treatment	Treatment
**Aghazadeh et al. 2020**	COVID-Confirmed. Severity NM	Eruption **preceded** to a typical COVID‐19 pneumonia.	In a weeks’ time	Treated conservatively with hydration, supplemental oxygen therapy at home
**Al-Khanati et al. 2020**	COVID-Confirmed. Severity NM	Aphthous ulcers appear at the **same time** as COVID-19 symptoms.	NM	NM
**Ansari et al. 2021**	COVID-Confirmed. Severity NM	**Case 1**: **Fifth day** after the onset of COVID-19 symptoms **Case 2**: **One-week** post-hospitalization	In about one week without scarring in both cases	Topical medications, (mixture of diphenhydramine, dexamethasone, tetracycline, and lidocaine)
**Brandão TB et al. 2021**	**Case 1&2**: Severe **Case 3,4,5,7 & 8**: Mild **Case 6**: Moderate	**Case 1**: 5^th^ day. **Case 2**: 4^th^ day. **Case 3**: 2^nd^ day. **Case 4**: 5^th^ day. **Case 5:** 10^th^ da**y.** **Case 6**^:^ 6^th^ day. **Case 7**: 8^th^ day **Case 8**: 8^th^ day of COVID symptoms.	**Case 1**: 11 days. **Case 2**: >15 days. **Case 3, 5 & 7**: 5 days. **Case 4**: 7 days **Case 6**^:^ 8 days. **Case 8**: 6 days	**Case 1 & 2**: HSV-1 detected. Initially, IV Acyclovir for 10 days, with no clinical improvement. Alternatively, for painful oral ulcers PBMT for another 10 days. **Case 2**: Lip ulcerations did not respond after 15 days of PBMT. **Case 3 & 4**: Patients receiving PBMT improved. **Case 5, 6, 7 & 8:** No treatment required.
**Cebeci K et al. 2020**	COVID-Confirmed. Severity NM	**Ten days** after the onset of COVID symptoms	Lesions resolved after few days of antibiotic therapy	Antibiotic therapy
**Chaux‑ Bodard A-G et al. 2020**	COVID-Confirmed. Severity NM	Ulcer **preceded** the COVID-19 confirmatory test.	After 10 days.	NM
**Ciccarese G et al. 2021**	Mild	About **5 days** from the onset of COVID-19 symptoms	After 5 days	IV immune globulins (400 mg/kg) and methylprednisolone (1 mg/kg)
**Corchuelo J et al. 2020**	Asymptomatic	**Three weeks** after COVID-19 confirmatory test	Lesion on the tongue resolved after two weeks of anti-fungal treatment. Time of resolution of ulcer NR	Nystatin for two weeks. Use of CHX 0.12%. Advised washing toothbrush in a NaOCl (1:100 dilution of 5% sodium hypochlorite) for 30 min, rinsing and drying before use.
**Cruz Tapia RO et al. 2020**	**Case 1&3**: (Mild) **Case 2**: (Hospitalized) **Case 4**: (NM)	**Case 1:** After some **8–9 days** of COVID-19 symptoms **Case 2**: NM **Case 3: 2**^**nd**^ **day** after COVID symptoms **Case 4**: **Same time** as COVID-19 symptoms	**Case 1:** NM **Case 2:** NM **Case 3:** After 5^th^ days **Case 4:** After 7^th^ days	**Case 1, 2, &3:** Nothing specific mentioned **Case 4:** Topical Cortisone solution and CHX 0.12% mouthwash
**Díaz Rodríguez M et al. 2020**	**Case 1**: Self isolated **Case 2&3**: Hospitalized	**Case 1**: **Last two weeks** of 56 days COVID symptoms duration. **Case 2**: Oral symptoms seen **after hospital discharge.** **Case 3**: **Since hospitalization** for COVID-19 symptoms	**Case 1:** Lingual de-papillation persisted but ulcers and burning sensation resolved. **Case 2**: Dysgeusia persists, oral lesion resolved. Cas**e 3**: Pseudomembranous lesions and commissural fissures resolved.	**Case 1**: Triamcinolone acetonide 0.05%, 3 times a day for 10‐days **Case 2**: Ointment (neomycin, nystatin, and triamcinolone acetonide) 3 times/d **Case 3**: Nystatin solution rinses; Ointment (neomycin, nystatin, and triamcinolone acetonide)
**Dominguez-Santas M et al. 2020**	COVID-Confirmed. Severity NM	**Case 1**: 4^th^ days. **Case 2**: 3^rd^ days. **Case 3**: 5^th^ days. **Case 4**: **same time** as COVID-19 symptoms	NM	NM
**dos Santos JA et al. 2020**	Severe	About **twenty-fourth days of hospitalization**	White lesion resolved after two -weeks. Geographic tongue condition improved from ‘severe’ to ‘moderate’ acc. to severity index after 54 days.	IV Fluconazole and oral nystatin. CHX (0.12%) alcohol-free mouth rinses with 1% hydrogen peroxide daily
**Favia G et al. 2021**	Confirmed Moderate, Severe and Critical COVID Cases	• In (n = 82; 65.9%) had oral lesions together with general COVID symptoms or within a week prior to any COVID therapy. • In seven cases blisters **preceded** ulcerative lesions • In 92% cases, **blisters and ulcerative lesions** appear either with general COVID symptoms or within one-week of the onset of COVID symptoms. • Petechiae often in association with angina bullosa appeared mostly after the initiation of COVID therapies. • In seven cases ulcero-necrotic gingivitis was observed in critical cases with poor oral hygiene.	Within 14 days	In patients with ulcero-erosive lesions Hyaluronic acid gel and 2% chlorhexidine mouthwash/gel (twice a day) for 14 days In patients with cytological diagnosis of candidiasis Miconazole Nitrate twice a day Tranexamic acid for local hemorrhages
**Glavina A et al. 2020**	Confirmed	About seven days after COVID-19 confirmatory test	After 3 weeks	Systemic Acyclovir therapy for five days. Local therapy (antiseptic, nystatin, panthenol, local anesthetic) for two weeks.
**Indu S et al. 2020**	Asymptomatic	**Same time** as the initial manifestation of COVID-symptom	After 10 days	NM
**Jimenez-Cauhe J et al. 2020**	COVID-Confirmed. Severity NM	Lesions appeared **after Hospital discharge.**	NM	Systemic corticosteroids
**Kämmerer T et al. 2021**	Severe	Three days after extubation from the ICU (secondary herpetic gingivostomatitis in the context of COVID‐19 infection)	NM	Oral acyclovir therapy 400 mg five times daily
**Kitakawa et al. 2020**	Confirmed Severity NM	At the **same time** as the initial manifestation of COVID-symptom	After 14 days	Nebacetin (Neomycin sulfate) ointment for 2 days
**Labé P et al. 2020**	**Case 1**: Confirmed **Case 2:** Suspected	**Case 1**: After 14 days of COVID-19 symptoms **Case 2**: NM	**Case 1**: After 2 weeks **Case 2**: NM	**Case 1**: NM **Case 2**: Intravenous gamma globulin
**Malih N et al. 2020**	COVID-Confirmed. Severity NM	Tonsillar aphthous lesion **precedes** COVID symptoms.	NM	Acetaminophen for pain control
**Martín Carreras- Presas C et al. 2020**	**Case 1**: Suspected **Case 2**: Suspected **Case 3**: Mild	**Case 1:** About 2–3 days. **Case 2:** NM **Case 3:** After a month.	**Case 1**: Resolved after 10 days. **Case 2**: 1 week. **Case 3**: After three days	**Case 1**: Valacyclovir 500 mg 3 times/d for 10 d, with topical CHX and hyaluronic acid. **Case 2**: Antiseptic mouthwash. **Case 3**: Hyaluronic acid and CHX mouthwash. prednisolone 30 mg/d
**Patel J et al. 2021**	Suspected	Oral symptoms appear **same time** as COVID-19 symptoms.	After 5-days	Metronidazole 400mg 3 times for 5 days and 0.12% chlorhexidine mouthwash twice daily for 10 days
**Sakaida T et al. 2020**	Severe	Erythematous lesions and erosions on the lips **preceded a week** before COVID-19 symptoms.	After 5-days	Oral prednisolone (20 mg/day)
**Soares CD et al. 2020**	COVID-Confirmed. Severity NM	NM	In three-weeks’ time	Dexamethasone, dipyrone
**Taşlıdere B et al. 2021**	Severe	COVID-19 infection can be considered among the causes of recurrence of the Melkersson–Rosenthal syndrome	NM	Hydroxychloroquine; Azithromycin, and steroid therapy
**Tomo S et al. 2020**	Confirmed Asymptomatic	About 9^th^ day of COVID-19 symptom	After 2 weeks	Chlorhexidine (0.12%) mouthwash

The majority of the oral lesions were painful. In addition, a case of a young female patient with symptoms of severe pruritus with painful, herpetic-like-lesion in the median lower lip area was also reported [30] (Table 2).

The collated cumulative data indicate, in general, oral lesions show no specific gender or age predilection. The common oral sites of presentation of the lesions, in descending order of prevalence, were the dorsum of the tongue, followed by the hard palate, soft palate, lips, buccal/labial mucosa, gingiva, tonsillar region, and the commissures (Table 2).

In terms of the temporality of presentation, oral mucocutaneous lesions preceded other COVID-19 symptoms in 2.4% (n = 4) of reported cases [17, 21, 32, 35], while in 51%, (n = 86) cases the lesions appeared simultaneously, or within a week of systemic SARS-CoV-2 symptoms [9, 12, 13, 18, 19, 22, 25–27, 30, 34]. Nonetheless, in some cases, this latency period stretched from two-to-three weeks after the onset of systemic COVID-19 symptoms [11, 24, 29, 31, 33, 37]. However, in two reported instances [24, 28], oral lesions emerged after the hospital discharge. Curiously, in a mild and asymptomatic case of COVID-19, the oral lesions manifested even after three weeks [23] and one month [33] after disease diagnosis (Table 3). Likewise, the time to resolution of the oral lesions also varied, from five days to two weeks. Few reports also documented persistent lingual de-papillation, dysgeusia, and geographic tongue even after the resolution of oral lesions following therapy for COVID-19 (Table 3).

**Table 3:** SARS-CoV-2 related oral mucocutaneous lesions clinical appearance, locations, and differential diagnosis.

Study	Location of oral muco-cutaneous lesion	Sign & Symptoms	Differential Diagnosis
**Aghazadeh N et al. 2020**	Lips, anterior tongue, and buccal mucosa	• Vesicular/herpetiform oral eruptions	Hand‐foot‐mouth disease; Atypical herpes simplex infection; Mycoplasma‐induced rash and mucositis; Erythema multiforme; Drug eruption
**Al-Khanati et al. 2020.**	Lower lip	• Aphthous‐like ulcers on the mucosa which became enlarged and painful over 3 days. • Burning sensation related to the tongue. • Halitosis.	NM
**Ansari R et al. 2021**	**Case 1**: Almost the entire hard palate **Case 2**: Anterior part of the tongue	**Case 1:** Painful ulcers of varying sizes; irregular margins, with red, non‐hemorrhagic background **Case 2:** Painful small ulcers; irregular margins with red, and non‐hemorrhagic background	Herpes simplex virus type 1 and type 2
**Brandão TB et al. 2021**	**Case 1 & 2**: Upper and lower lip mucosa, anterior dorsal tongue **Case 3**: Lateral border of tongue and anterior hard palate **Case 4**: Upper and lower lip **Case 5**: Apex and lateral borders of the tongue **Case 6**: Tonsillar pillar **Case 7**: Ventral portion of the tongue **Case 8**: Upper and lower labial mucosae and lateral border of the tongue	**Case 1, 3 & 4:** Painful aphthous like ulcers with necrosis **Case 2**: Painful hemorrhagic ulcers **Case 5–8:** Painful aphthous like ulcers **Case 6:** Hemorrhagic ulcers with necrotic areas	NM
**Cebeci K et al. 2020**	Oropharynx, hard and soft palate	• Erythematous surface, few petechiae, numerous pustular enanthema	NM
**Chaux‑Bodard A-G et al. 2020**	Dorsal surface of the tongue	• Initially, painful inflammation, followed by painful erythematous macula, then an asymptomatic irregular ulcer	NM
**Ciccarese G et al. 2021**	Lips (inner surface), palate and gingiva	• Erosions, ulcerations, and blood crusts on the inner surface of the lips. • Palatal and gingival petechiae	NM
**Corchuelo J et al. 2020**	Lower lip, attached gingiva of the lower left first premolar, and tongue	• Multiple painless petechiae on the lower lip. • Whitish area at the back of tongue. • Painful aphthous ulcer on the attached gingiva of premolar	Whitish area on the tongue suggestive of *Candidiasis*.
**Cruz Tapia RO et al. 2020**	Hard palate, tongue	**Case 1&3**: asymptomatic non-bleeding purple bulla (hard palate & tongue, respectively). **Case 2**: macule and papule‐plaque (hard palate). **Case 4:** Erythematous small macules (hard palate)	Angina bullosa hemorrhagic‐like lesion
**Díaz Rodríguez M et al. 2020**	Tongue, palate, and commissure	**Case 1**: aphthous‐like lesions with tongue de-papillation **Case 2**: burning mouth sensation and unilateral commissural fissures. **Case 3**: lesions compatible with pseudomembranous candidiasis and angular cheilitis	NM
**Dominguez-Santas M et al. 2020**	Buccal and labial mucosa; mucogingival junction; ventral surface of tongue	**Case 1 to 4**: single to cluster of aphthous ulcers	Herpes simplex virus, Epstein‐Barr virus, and Cytomegalovirus
**dos Santos JA et al. 2020**	Tongue	• White plaque, with multiple pinpoint yellowish ulcers on the dorsum of the tongue dorsum.	Oral candidiasis; Herpetic recurrent oral lesions
**Favia G et al. 2021**	Tongue, hard palate, lip, buccal mucosa, soft palate, gingiva	**Moderate COVID**: Geographic tongue (5); Fissured tongue (4); Painful ulcerative lesion (51); Blisters (14); Hyperplasia of papillae (33); Angina bullosa (8); Candidiasis (18); Ulcero-necrotic gingivitis (1); Petechiae (4) **Severe COVID**: Geographic tongue (2); Fissured tongue (1); Painful ulcerative lesion (11); Blisters (5); Hyperplasia of papillae (13); Angina bullosa (2); Candidiasis (4); Ulcero-necrotic gingivitis (2); Petechiae (6) **Critical Cases**: Painful ulcerative lesion (3); Hyperplasia of papillae (2) - Angina bullosa (1); Candidiasis (6); Ulcero-necrotic gingivitis (4); Petechiae (4); Spontaneous oral hemorrhage (1)	NM
**Glavina A et al. 2020**	Lip, hard palate, and tongue	• Herpetic vesicles on the lips and hard palate • White hairy tongue and non-specific white lesion on the ventral surface of the tongue • Pain and burning sensation in the oral cavity	NM
**Indu S et al. 2020**	Labial mucosa and ventral surface of the tongue	• Unilateral, painful, shallow, round to oval shape ulcers surrounded by an inflammatory halo	Herpes Zoster infection
**Jimenez-Cauhe J et al. 2020**	Palate	Macules and petechiae	NM
**Kämmerer T et al. 2021**	Oral mucosa	Multiple, sharply circumscribed, painful ulcerations covered by yellow–grey membranes	Herpes simplex virus (HSV)‐1/2 antibodies, Behçet disease (have no prior history of Herpes)
**Kitakawa et al. 2020**	Lip	A herpetic like-lesion in the median lower lip semi-mucosa, with severe pruritis	Recurrent Herpes
**Labé P et al. 2020 et al. 2020**	Lip, oral mucosa, tongue	**Case 1**: Severe painful, erosive cheilitis, diffuse gingival erosions with thick hemorrhagic crusts **Case 2**: Painful cheilitis and glossitis	**Case 1**: Herpes simplex virus (HSV) **Case 2**: COVID‐19‐associated Kawasaki disease
**Malih N et al. 2020**	Tonsillar region	Erythema and painful aphthous lesion on the left tonsil	Herpes simplex lesions
**Martín Carreras- Presas C et al. 2020**	**Case 1 & 2**: Hard palate **Case 3**: Inner lip mucosa and gingiva	**Case 1**: Painful, orange‐colored ulcers with an erythematous halo. **Case 2**: Multiple unilateral, painful, pinpoint yellowish ulcers with an erythematous halo. **Case 3**: Painful blisters on lip mucosa and desquamative gingivitis	**Case1 & 2:** Herpes simplex lesions **Case 3**: Erythema multiforme
**Patel J et al. 2021**	Gingiva in both the maxillary and mandibular labial sextants	• Severe halitosis with generalized erythematous and edematous gingivae. • Necrotic interdental papillae. • Gingival sulcus bleeding without any provocation	Necrotizing gingivitis
**Sakaida T et al. 2020**	Lower lip and buccal mucosa	Erythematous lesions and erosions	NM
**Soares CD et al. 2020**	Buccal mucosa, hard palate, tongue, and lips	Painful, scattered, ulcerated lesion, reddish macules of varying sizes	NM
**Taşlıdere B et al. 2021**	Face, lips, and tongue	• Edema in the right lower lip • Right facial paralysis • Fissured tongue	Cytomegalovirus; Herpes simplex virus, Epstein–Barr virus; Coxsackie virus infection
**Tomo S et al. 2020**	Tongue and soft palate	• Oral mucositis characterized by diffuse, bilateral erythema with de-papillation in the borders of the tongue. • Burning sensation in the borders of the tongue and soft palate	NM

Depending on the type of lesion, a wide range of therapeutic measures was employed for their management (Table 3). These included chlorhexidine mouthwashes, topical or systemic corticosteroids, antibiotics, antifungals, antiviral drugs alone or in combination with antibiotics, and drugs for pain relief. In one case, a young 9-year-old female patient’s vesicular herpetic eruptions resolved approximately a week after conservative treatment using only hydration therapy [17]. Interestingly, photo-biomodulation (PBMT) therapy was employed for pain management of oral ulcers in few other cases [9] (Table 3).

## Discussion

The receptor-binding domain of SARS-CoV-2 has an intense affinity for ACE_2_ -functional receptors [38]. The ubiquitous presence of ACE_2_ in the lining mucosa of the nose, lung, pharynx, oral mucosa, and salivary glands, as well as multiple organ systems of humans, make them not only essay portals of viral access to the body but also vulnerable to viral damage [39]. The varied oral-facial mucocutaneous manifestations of COVID-19 reported in the literature and reviewed here could, therefore, be due either to the primary, focal damage caused by the virus at the point of tissue entry, and/or a reflection of secondary damage due to the generalized effect of systemic SARS-CoV-2 infection and the ensuing immunologic abnormalities [4, 8]. We discuss our findings under two major categories, generalized oral manifestations, and muco-cutaneous manifestations. The former, which addresses i) gustatory dysfunction ii) xerostomia, and iii) burning mouth, is included in the narrative as they are intimately linked to mucocutaneous disease.

### Generalized oral manifestations

#### Gustatory dysfunction

From the beginning of the SARS-CoV-2 pandemic, multiple reports from several regions of the world indicated that aberrations of taste sensation either as dysgeusia or ageusia were particularly common in diseased individuals and presented mainly as a premonitory or an early symptom of the disease [40, 41] (Fig 2). The studies reviewed here corroborate these data from the early stage of the pandemic and indicate that either the total acute loss of taste–ageusia, or alterations in the taste sensation–dysgeusia or hypogeusia, are a frequent symptom of SARS-CoV-2 infection. However, contradicting the previous literature [6, 42, 43], we found no specific gender predilection in the alterations of taste sensation in the collated and reviewed data.

A number of postulates have been proposed for the pathogenesis of the gustatory disorders associated with COVID-19. Some contend that ACE_2_ receptors on lingual keratinocytes and supporting cells of taste buds are the first to be infected, leading to virus-induced cell dysfunction and death, and subsequent alterations of taste perception [44]. A postmortem report by Favia et al. (2021) supports this hypothesis. They provide histopathological data of lingual papillae of COVID-19 patients with enlarged, inflamed papillae of the lining mucosa and heavy monocytic/ lymphocytic infiltrates, as well as vascular hyperplasia in tissues subjacent to taste buds [12]. Furthermore, it is noteworthy that ACE_2_ expression is higher in the tongue than in other oral mucosal tissues [45].

Loss of smell or anosmia is simultaneously present in most COVID-19 patients with dysgeusia/ageusia [40, 41]. Hence some have surmised that, as gustatory and olfactory sensations are closely linked, the viral damage to the olfactory epithelium may either fully or partly account for the acute onset ageusia [46, 47]. Indeed, profuse expression of ACE_2_ receptors is seen in the olfactory neuroepithelium, that are infected during COVID-19.

Other explanations for dysgeusia have been offered, particularly when it persists after the initiation of COVID-19 therapy and after hospital discharge. These include afflictions of the peripheral nervous system innervating the taste buds [48] and the possible side effects of medications such as antibiotics, corticosteroids, and immunosuppressants, prescribed for managing COVID-19 [48, 49].

#### Xerostomia

In the reviewed reports, gustatory dysfunction (dysgeusia/hypogeusia) was present either as the sole oral manifestation or associated with xerostomia and/or burning mouth sensation in 69.8 percent of the patients (Fig 2) In a recent report, Huang et al., using single-cell RNA (scRNA) analyses, elegantly illustrated the rich expression of ACE2 and transmembrane serine protease (TMPRSS) receptors for SARS-CoV-2 in the acinar and duct epithelial cells of both the major and minor salivary glandular tissue derived from COVID-19 patients [5]. Such salivary gland invasion by SARS-CoV-2 may have two significant consequences: first, the infection *per se* may affect the functionality, and the quality and the quantity of the salivary secretions, and second, saliva could, coincidentally, serve as a rich source of infectious particles promoting the spread of infection.

Interestingly, one report described a COVID-19 patient who complained of extremely viscous saliva [11]. Therefore, it is tempting to speculate that the latter manifestation could be due to the primary viral infection affecting the glandular tissue of the major, serous salivary gland (parotid), leading to the predominantly mucus/viscous secretions [50]. Nevertheless, the pathobiology of xerostomia secondary to the SARS-CoV-2 infection remains to be determined through further studies.

As mentioned above, a few of the reviewed cases simultaneously presented with both dysgeusia and xerostomia [10, 11, 37]. Physiologically, the taste sensation is a critical central stimulant for initiating salivary secretions [51]. It could, therefore, be postulated that gustatory dysfunction noted in a vast proportion of COVID-19 patients could be bimodal in nature: primarily neuronal, affecting the physiology and functionality of the taste buds due to the well-known neuro-tropic and invasiveness of the virus [52], and a secondary effect due to the coincidental xerostomia. However, more data are needed to ascertain the relationship between xerostomia and salivary gland dysfunction in COVID-19.

#### Burning mouth

It is known that the burning mouth sensation usually presents in a majority of patients with either dysgeusia and/or xerostomia [53, 54]. Yet, the burning mouth sensation is a rather non-specific disease entity related to a multiplicity of other conditions, including psychiatric disorders, candidal infections, diabetes, various drugs, vitamin and/or mineral deficiencies [53]. Though not specifically recorded, one or more of these may have been etiologically involved in the reviewed cohort of patients who complained of burning mouth. For instance, it is noteworthy that a few with burning oral sensations showed signs of candidal infection [12, 24].

### Oral mucocutaneous manifestations

Viral infections in the oro-facial regions are relatively common. They mostly manifest as maculopapular lesions and ulcers of various shapes and sizes, in addition to neurologic manifestations, including facial palsies [55]. Herpes group viruses, such as herpes simplex, herpes zoster, cytomegalovirus, Epstein-Barr virus, are the commonest viruses that infect the oro-facial region [55, 56]. Recrudescence of viral infections, mostly due to the herpes group of viruses, may also occur as secondary diseases, due to generalized immunosuppression consequential to radiotherapy, cytotoxic or steroid drugs, or in systemic infections such as the human immunodeficiency virus (HIV) disease that incapacitates the immune system in general [57]. Additionally, viruses such as the human papillomavirus (HPV), present as innocuous passengers in the salivary virome of a high proportion of individuals [58], may also induce dysplastic and neoplastic changes of the mucosal epithelium, as well [59].

Whilst the foregoing viruses are common agents affecting the oro-facial region, a string of novel viruses that cause respiratory tract infections in humans, including Severe Acute Respiratory Syndrome Coronavirus– 1(SARS-CoV-1), Middle East Respiratory Syndrome Coronavirus (MERS‐CoV), and H7N9 influenza A virus, a swine‐like influenza H3N2 variant virus, and a human adenovirus 14p1, are now known to affect the oral cavity with ill-defined, nondescript manifestations [60]. Furthermore, it now appears that the newest addition to this list is SARS-CoV-2 that may induce both primary and secondary oral infections, as noted in the reviewed publications.

Clearly, the oro-pharyngeal region appears to be a major reservoir of the SARS-CoV-2 in the infected cohorts [4]. In addition, molecular studies indicate the profuse expression of ACE_2_ receptors in the epithelia of the dorsal lingual surface, hard palate, soft palate, lips, buccal, and labial mucosa [4, 39]. This was reflected by the predominance of mucocutaneous lesions in these anatomical locales. The lesions, in general, had a nondescript expression profile, ranging from aphthous-like ulcerations, erosions, mucositis, pigmentations, hemorrhagic crusts, desquamative gingivitis, as well as cheilitis. It is, however, difficult to state with any degree of certainty whether the lesions were due primarily to SARS-CoV-2 infection or a secondary effect consequential to the general debility and/or drug therapy, or possible immune dysfunction seen in these patients.

A case in point is the enanthems observed by some that were categorized as petechial, erythematous, vesicular, macular, or macular with petechiae [4, 61]. This is in contradistinction to enanthems in other viral infections such as herpangina, measles, human herpesvirus (HHV) infections, and roseola infantum that present with specific, pathognomonic characteristics identifiable with the disease entity [61]. Thus, for example, enanthem in herpangina appears as small vesicular or ulcerative lesions confined to the posterior oropharyngeal region, or in the case of roseola infantum, ’Nagayama spots’ presenting as erythematous papules on the soft palate and at the base of the uvula [62].

#### Oral ulcers

Oral ulcers were the most frequent lesion reported in the reviewed COVID-19 cases. The pathogenesis of these lesions remains speculative at present. Disruption of the superficial epithelial barrier of the oral mucosa due either to viral invasion and/or the collateral damage due to the host immune responses against the viral antigenic components of the mucosal epithelium is yet to be resolved. For instance, increased level of tumor necrosis factor (TNF)-α noted in COVID-19 infection [63] may lead to resultant IL-1, and IL-6 production and chemotaxis of inflammatory cells into the viral-laden epithelium, which in turn may cause apoptosis, necrosis and consequent ulceration [64, 65]. The work of Favia et al. [12] lends some credence to this notion, as they observed complete disruption of the epithelial covering, vascular hyperplasia, perivascular hemorrhage, and lymphomonocytic infiltrations in the sub-epithelial tissues in the central necrotic area of the ulcerated regions, in addition to thrombosis of small and medium-sized vessels in subjacent tissues. Histopathological observations of oral mucosal lesions in COVID-19 by other workers also attest to the presence of inflammatory infiltrates in the affected epithelium [10, 19, 28].

#### Angina bullosa and petechial lesions

The exaggerated inflammatory response evoked by SARS-CoV-2 is now recognized as a harbinger of vascular endothelial dysfunction and subsequent thrombus formation [66, 67]. Hence, anticoagulants remain an essential management tool for the thromboembolic phenomena seen in COVID-19 patients [67]. Therefore, petechiae and angina bullosa in the oral mucosa reported in several studies [12] could conceivably be linked to such anticoagulant therapies. Furthermore, in several reports, petechial spots were observed on the hard and soft palate, lips, and the oropharynx [12, 22, 24, 25, 30]. However, as these reports did not indicate whether the patients were undergoing anticoagulant therapy prior to such presentation, further studies are required to confirm or refute the aforesaid contention.

#### Oral mycoses

The portrayal of an asymptomatic COVID-19 patient with oral candidiasis by Corchuelo and Ullioa (2020) [23] brings to light another facet of COVID-19 manifestation. The latter group opined that generalized immunosuppression might lead to opportunistic fungal infections even in the absence of overt signs of COVID-19. A subsequent report has described two COVID-19 patients with esophageal candidiasis but with no history of any antibiotic or immunosuppressant use [68]. Nevertheless, they too surmised that the profound and acute T-cell immunologic deficit due to SARS-CoV-2 infection might have led to the fungal infections [68].

Another multicenter study by Salehi and colleagues (2020) [69] reported the risk of opportunistic fungal infection in ventilation supported COVID-19 patients with respiratory distress syndrome, receiving broad-spectrum antibiotics and/or corticosteroids, both of which are well known to cause oral candidiasis [70]. Others have also published reports of hospitalized COVID-19 patients having oral candidiasis, which improved with antifungal medication [11, 12, 24]. Interestingly, a case series of COVID-19 patients with angular cheilitis, known to be due to mixed candidal-bacterial infection [71] is also noteworthy in this context [72].

Incidentally, mucormycosis, colloquially known as `black fungus disease,`is a relatively common presentation, mainly reported from the Indian sub-continent [73], and may present as oral ulcerations in post-COVID-19 states after recovery [74, 75]. However, our reviewed reports did not encompass any cases of oral mucormycosis, possibly due to its very recent recognition in COVID-19 patients, particularly in the South Asian region. Apart from candidiasis and mucormycosis, the third common fungal disease reported in COVID-19 patients is pulmonary aspergillosis [76]. Yet again, we did not note any oral mucocutaneous lesions caused by this fungus in the current review. The foregoing strongly suggests the not uncommon prevalence of opportunistic oral fungal coinfections superimposed on systemic viral disease, not unlike that seen in HIV infection, where oral candidiasis is a predominant early manifestation [77].

Taken together, these observations support the hypothesis that oral ulcerations in COVID-19 may be due to a triad of effects, first, as a direct result of the viral damage to the epithelium, second, an indirect effect of the immune dysregulation of the mucosal epithelium, and third due to the underlying therapeutic regimens such as corticosteroids used in the disease management. However, deciphering the role of each of these entities need to be performed when a more extensive database of COVID-19 patients is available, and when such cohorts are followed up for a significant period.

### Periodontal health

Regarding the periodontal health of the reviewed cohort, we noted in some individuals the relatively uncommon condition of necrotizing ulcerative gingivitis [12, 34, 78] (Fig 2). This is a painful and precipitously progressive disease involving free/attached gingivae and the alveolar mucosa, with ulceration, pain, necrosis, and bleeding. The condition may occur as a complication of chronic gingivitis when oral hygiene is neglected, especially in debilitated individuals, and/or when the systemic immune system is compromised, as in the case of HIV disease [79]. In general, the condition was documented in several critically ill COVID-19 patients with poor oral hygiene, and further, follow-up studies are required to ascertain the pathogenesis of such lesions.

### Treatment algorithms

A well-recognized, standard therapeutic algorithm for managing the oral manifestations of COVID-19 is unavailable, as yet, most likely due to the novelty of the disease entity. This, not surprisingly, has led to a variety of management regimens in different jurisdictions (Table 2). Most of the reviewed cases were managed by topical steroids alone or combined with antibacterial and antivirals, mainly as ingredients in ointments (Table 2). For instance, Glavina et al. (2020) [26] reports systemic Acyclovir combined with a topical antiseptic, a polyene antifungal nystatin, panthenol (a skin antiseptic), as well as a local anesthetic for treating painful herpetic vesicles, and non-specific white lesions in a 40-years old female. Likewise, Ansari et al. (2021) [19] and Diaz Rodríguez et al. (2020) [24] used topical ointment with an assortment of anti-fungal, antibacterial, and steroidal suspensions with painkillers to manage non-specific complaints resembling candidiasis on the lingual surface. On the contrary, in a major departure from the conventional treatment, Brandão et al. (2021) used photo biomodulation therapy (PBMT) for managing painful oral ulcers, with some degree of success [9]. In two reports, ulcero-erosive lesions were treated using hyaluronic acid gel with chlorhexidine [12, 33]. The former also used tranexamic acid to arrest bleeding in ulcers [12].

Stand-alone therapies used by clinicians include the administration of systemic antibiotics by Cebeci et al. (2020) for treating pustular oral manifestation [20] and the application of neomycin ointment to treat herpetiform and zosteriform lesions [30]. Jimenez-Cauhe (2020) used systemic corticosteroids for erythema multiform-like manifestations that appeared in a severe case of COVID following hospital discharge [28]. Others have used various conservative techniques for the management of oral manifestations, such as home-based hydration and supplemental oxygen therapy [17] or chlorhexidine mouthwash alone [37].

The foregoing, wide, and disparate variety of medications used to treat oral manifestations of COVID-19 testifies to the urgent need for a well-formulated, consensus therapeutic regimentation for managing oral manifestations of COVID-19.

### Data quality and limitations

The critical appraisal of the reviewed data indicates that most reports could be categorized as having a low to moderate risk of biases (Table 1). We noted, for instance, deficiencies in the self-assessment reports of subjectively perceived taste disorder symptoms. The accuracy of such self-diagnosed symptomatology can introduce intrinsic response biases unless confirmed by an experienced clinician using standardized and validated instruments. Also, some authors did not explicitly state the degree of severity of COVID-19 of their patients. Furthermore, most case reports of mucosal lesions were devoid of histopathological information despite categorization as a specific disease entity.

## Conclusions

Oral manifestations of COVID-19 patients encompassing the current review varied considerably for a number of reasons. Apart from those mentioned above, these included the non-specific reference point descriptors. For instance, in several reports, hospital admission, treatment commencements, hospital discharge, other respiratory and systemic manifestations are utilized as reference points to describe the onset of oral signs and symptoms, leading to discrepant temporal associations of COVID-19 onset and oral manifestations. This, notwithstanding, our review highlights that in over half of the COVID-19 cases, oral mucosal manifestations either precede or appear simultaneously with symptoms of SARS-CoV-2 infection, suggesting an association, and not necessarily causation, between the viral infection and oral disease. Furthermore, apart from the now well-known taste disorders in COVID-19 [80], the pathogenesis of mucocutaneous lesions, such as candidiasis and hemorrhagic lesions (petechiae/angina bullosa), is likely to be secondary to antimicrobial or steroidal or anticoagulant therapies, and/or the COVID-19 associated immune impairment, rather than a direct result of SARS-CoV-2 infection *per se*. Nevertheless, prompt recognition and management of oral mucocutaneous manifestations are essential to improve the quality of life of these patients. Last but not least, the proper care and the management of the varied oral manifestations, particularly in the severe and hospital-bound COVID-19 patients should be performed by a multispecialty team including dental practitioners versed in oral medicine and pathology.

## Supporting information

S1 Checklist PRISMA 2020 checklist. (DOCX)

S1 File Table -JBI checklist for case report. (DOCX)
