# Supplementary material for: Oro-facial mucocutaneous manifestations of Coronavirus Disease-2019 (COVID-19): A systematic review
Source: PLoS One. 2022 Jun 1;17(6):e0265531. doi: 10.1371/journal.pone.0265531 (PMC9159624; doi:10.1371/journal.pone.0265531)
Supplement: S1 File — (DOCX) [file pone.0265531.s002.docx]

**Supplementary file: Table -JBI Checklist for Case Report**

| **Study** | **Q1** | **Q2** | **Q3** | **Q4** | **Q5** | **Q6** | **Q7** | **Q8** | **Overall score** |
| --- | --- | --- | --- | --- | --- | --- | --- | --- | --- |
| **Aghazadeh N et al. 2020** | 1 | 1 | 1 | 0 | 1 | 1 | 0 | 1 | 6 |
| **Al Khanati et al. 2020.** | 0 | 1 | 1 | 1 | 1 | 0 | 0 | 1 | 5 |
| **Ansari R et al. 2020** | 1 | 1 | 1 | 1 | 1 | 1 | 0 | 1 | 7 |
| **Cebeci K et al. 2020** | 1 | 0 | 1 | 1 | 1 | 1 | 1 | 1 | 7 |
| **Chaux‑ Bodard A-G et al. 2020** | 1 | 0 | 1 | 1 | 1 | 0 | 0 | 1 | 5 |
| **Ciccarese G et al. 2020** | 0 | 1 | 1 | 1 | 1 | 1 | 0 | 1 | 6 |
| **Corchuelo J et al. 2020** | 1 | 1 | 1 | 0 | 1 | 1 | 1 | 1 | 7 |
| **Cruz Tapia RO et al. 2020** | 0 | 1 | 1 | 1 | 1 | 0 | 0 | 1 | 5 |
| **Díaz Rodríguez M et al. 2020** | 1 | 0 | 1 | 1 | 1 | 0 | 0 | 1 | 5 |
| **Dominguez-Santas M et al. 2020** | 0 | 1 | 1 | 1 | 1 | 1 | 1 | 1 | 7 |
| **dos Santos JA et al. 2020** | 1 | 1 | 1 | 0 | 1 | 1 | 0 | 1 | 6 |
| **Glavina A et al. 2020** | 1 | 1 | 1 | 1 | 1 | 1 | 1 | 1 | 8 |
| **Indu S et al. 2020** | 0 | 1 | 1 | 1 | 1 | 0 | 0 | 1 | 5 |
| **Jimenez-Cauhe J et al. 2020** | 1 | 0 | 1 | 1 | 1 | 0 | 0 | 1 | 5 |
| **Kämmerer T et al. 2021** | 0 | 1 | 1 | 1 | 1 | 1 | 0 | 1 | 6 |
| **Kitakawa et al. 2020** | 1 | 1 | 1 | 1 | 1 | 1 | 1 | 1 | 8 |
| **Labé P et al. 2020** | 1 | 0 | 1 | 1 | 1 | 0 | 0 | 1 | 5 |
| **Malih N et al. 2020** | 0 | 1 | 1 | 1 | 1 | 1 | 0 | 1 | 6 |
| **Martín Carreras‑ Presas C et al. 2020** | 0 | 1 | 1 | 1 | 1 | 0 | 1 | 1 | 6 |
| **Patel J et al. 2020** | 1 | 1 | 1 | 0 | 1 | 1 | 1 | 1 | 7 |
| **Sakaida T et al. 2020** | 1 | 1 | 1 | 1 | 1 | 1 | 0 | 1 | 7 |
| **Soares CD et al. 2020** | 1 | 1 | 1 | 0 | 1 | 1 | 0 | 1 | 6 |
| **Taşlıdere B et al. 2021** | 0 | 1 | 0 | 1 | 1 | 1 | 1 | 1 | 6 |
| **Tomo S et al. 2020** | 0 | 1 | 1 | 0 | 1 | 1 | 1 | 1 | 6 |

5/8=63%; 6/8=75%; 8/8=100%

**JBI Case Series**

| **Study** | **Q1** | **Q2** | **Q3** | **Q4** | **Q5** | **Q6** | **Q7** | **Q8** | **Q9** | **Q10** | **Overall score** |
| --- | --- | --- | --- | --- | --- | --- | --- | --- | --- | --- | --- |
| **Brandão TB et al. 2021** | **1** | **1** | **1** | **1** | **1** | **0** | **1** | **1** | **1** | **1** | **9** |

9/10= 90%

**JBI Cross sectional study (Observational study)**

| **Study** | **Q1** | **Q2** | **Q3** | **Q4** | **Q5** | **Q6** | **Q7** | **Q8** | **Overall score** |
| --- | --- | --- | --- | --- | --- | --- | --- | --- | --- |
| **Favia G et al. 2021** | 1 | 1 | 1 | 1 | 0 | 0 | 1 | 1 | 6 |

6/8= 75%
